# A novel positive feedback-loop between the HTLV-1 oncoprotein Tax and NF-κB activity in T-cells

**DOI:** 10.1186/s12977-020-00538-w

**Published:** 2020-09-10

**Authors:** Sebastian Millen, Lina Meretuk, Tim Göttlicher, Sarah Schmitt, Bernhard Fleckenstein, Andrea K. Thoma-Kress

**Affiliations:** Institute of Clinical and Molecular Virology, Universitätsklinikum Erlangen, Friedrich-Alexander-Universität Erlangen-Nürnberg, Erlangen, Germany

**Keywords:** HTLV-1, Tax-1, NF-κB, M22, Protein stability, IKK2

## Abstract

**Background:**

Human T-cell leukemia virus type 1 (HTLV-1) infects primarily CD4^+^ T-lymphocytes and evoques severe diseases, predominantly Adult T-Cell Leukemia/ Lymphoma (ATL/L) and HTLV-1-associated Myelopathy/ Tropical Spastic Paraparesis (HAM/TSP). The viral transactivator of the pX region (Tax) is important for initiating malignant transformation, and deregulation of the major signaling pathway nuclear factor of kappa B (NF-κB) by Tax represents a hallmark of HTLV-1 driven cancer.

**Results:**

Here we found that Tax mutants which are defective in NF-κB signaling showed diminished protein expression levels compared to Tax wildtype in T-cells, whereas *Tax* transcript levels were comparable. Strikingly, constant activation of NF-κB signaling by the constitutive active mutant of *inhibitor of kappa B kinase* (IKK2, IKK-β), IKK2-EE, rescued protein expression of the NF-κB defective Tax mutants M22 and K1-10R and even increased protein levels of Tax wildtype in various T-cell lines while *Tax* transcript levels were only slightly affected. Using several Tax expression constructs, an increase of Tax protein occurred independent of *Tax* transcripts and independent of the promoter used. Further, Tax and M22 protein expression were strongly enhanced by 12-O-Tetradecanoylphorbol-13-Acetate [TPA; Phorbol 12-myristate 13-acetate (PMA)]/ ionomycin, inducers of NF-κB and cytokine signaling, but not by tumor necrosis factor alpha (TNF-α). On the other hand, co-expression of Tax with a dominant negative inhibitor of κB, IκBα-DN, or specific inhibition of IKK2 by the compound ACHP, led to a vast decrease in Tax protein levels to some extent independent of *Tax* transcripts in transiently transfected and Tax-transformed T-cells. Cycloheximide chase experiments revealed that co-expression of IKK2-EE prolongs the half-life of M22, and constant repression of NF-κB signaling by IκBα-DN strongly reduces protein stability of Tax wildtype suggesting that NF-κB activity is required for Tax protein stability. Finally, protein expression of Tax and M22 could be recovered by NH_4_Cl and PYR-41, inhibitors of the lysosome and the ubiquitin-activating enzyme E1, respectively.

**Conclusions:**

Together, these findings suggest that Tax’s capability to induce NF-κB is critical for protein expression and stabilization of Tax itself. Overall, identification of this novel positive feedback loop between Tax and NF-κB in T-cells improves our understanding of Tax-driven transformation.

## Background

Human T-cell leukemia virus type 1 (HTLV-1) is an oncogenic delta-retrovirus that persistently infects around 5–10 million people worldwide [1]. HTLV-1 causes Adult T-cell Leukemia/ Lymphoma (ATL/L), HTLV-1 associated Myelopathy/ Tropical Spastic Paraparesis (HAM/TSP) and other inflammatory diseases in up to 10% of infected individuals [2–4]. Endemic areas are southwestern regions of Japan, South America, Sub-Saharan Africa, the Caribbean and parts of the Middle East, Australia and Melanesia [1]. HTLV-1 infects mainly CD4^+^ T-cells and, to a lesser extent, CD8^+^ T-cells, monocytes, and dendritic cells [5–7], however, persistent infection, transformation and immortalization preferentially occurs in CD4^+^ T-cells. HTLV-1 is transmitted via body fluids containing infected cells, like breast milk, semen, blood products, or organ transplants [8, 9]. Upon infection and reverse transcription, HTLV-1 integrates into the host genome, persisting mainly in its proviral form. Next to typical retroviral structural and enzymatic proteins, HTLV-1 encodes regulatory (Tax, Rex) and accessory (p8/p12, p13, p30, HBZ) proteins [10]. Amongst these, Tax plays a crucial role in cell-to-cell transmission as well as cellular transformation.

Tax is a powerful and multifaceted protein. It comprises of 353 amino acids (aa) and contains several defined domains which allow it to interact with a multitude of cellular factors and regulators [10–14]. It not only initiates transcription from the HTLV-1 long terminal repeat (LTR) regions via Tax responsive elements (TREs) but also serves as a transcriptional activator of cellular pathways like cAMP response element-binding protein/ Activating transcription factor (CREB/ATF) and Activator protein 1 (AP-1) [15–17]. Importantly, Tax is a prototypic oncogene as Tax alone is sufficient to immortalize T-cells in vitro [18–22]. It was shown that CREB/ATF and Serum response factor (SRF) signaling are involved in cellular transformation, however, nuclear factor of kappa B (NF-κB) is the major pathway deregulated in order to achieve cellular transformation [23].

NF-κB is a family of transcription factors that regulates gene expression as a response to a multitude of extra- and intracellular stimuli [24–26]. It consists of five structurally similar DNA-binding proteins, RelA (p65), RelB, c-Rel, NF-κB1 (p50 and p105), and NF-κB2 (p52 and p100). Two distinct NF-κB pathways are described, the canonical and non-canonical or alternative NF-κB pathway [27]. Canonical NF-κB as prototypic NF-κB signaling is stimulated by various extra- and intracellular stimuli, including tumor necrosis factor alpha (TNF-α), oxidative stress or bacterial infections, and regulates inflammatory responses [28]. On the other hand, non-canonical NF-κB signaling regulates lymphoid organogenesis and adaptive immunity, being stimulated by CD40L or B-cell activating factor (BAFF) [29, 30]. Tax of HTLV-1 is known to activate both NF-κB signaling pathways. In the nucleus and cytoplasm, Tax interacts with the inhibitor of kappa B kinase (IKK) regulatory subunit IKK-γ [IKK-3; NF-κB essential modulator (NEMO)], thereby triggering subsequent NF-κB activatory steps including phosphorylation of inhibitor of kappa B (IκB) and RelA [31, 32]. In the nucleus, Tax associates with RelA and accumulates in Tax nuclear bodies, representing foci of maximal transcriptional activity [33, 34]. As a result, activation of canonical NF-κB signaling eventually leads to induction of p100 protein, while activation of non-canonical NF-κB induces proteolytic degradation of p100 to p52. Counteracting Tax’s activatory role, the HTLV-1 anti-sense protein basic zipper factor (HBZ) dampens NF-κB activity by preventing Tax-induced cellular senescence and by interfering with p65 protein functions [35, 36]. As Tax itself most likely does not exert kinase functions, it relies on recruitment of several kinases, like transforming growth factor (TGF) beta-activated kinase 1 (TAK1), NF-κB inducing kinase (NIK), or mitogen-activated protein kinase kinase kinase (MAP3K) in a TNF receptor-associated factor (TRAF) 3-dependent manner in order to activate the IKK complex, which consists, next to the regulatory platform subunit NEMO, of the catalytic subunits IKK1 (IKK-α) and IKK2 (IKK-β) [37–40]. Interestingly, apart from being recruited by Tax activity, NIK has been demonstrated to be activated by epigenetic regulation [41]. Essentially, Tax protein is object of massive post-translational modifications (PTMs), including Tax phosphorylation, small ubiquitin-related modifier conjugation (SUMOylation) and ubiquitinylation, which not only regulate Tax’s subcellular localization but also its protein–protein interactions [42]. There is strong evidence that ubiquitinylation of Tax is the major PTM essential for Tax-mediated activation of NF-κB as Tax employs several ubiquitin conjugating (Ubc) and ubiquitin ligating enzymes, like Ubc-13 and the ring finger proteins (RNF) 4 and 8 in order to activate TAK1 and the IKK complex [39, 43–45]. Additionally, Tax’s impact on NF-κB might be further finetuned by activity of the ubiquitinylation-dependent sequestosome 1 (SQSTM1/p62), including the autophagy pathway [46].

The requirement of NF-κB activation by Tax for cellular transformation has not conclusively been resolved. On the one hand, the NF-κB deficient Tax mutant S258A was still able to transform human primary T-lymphocytes [47, 48]. On the other hand, most studies imply a critical role of NF-κB in transformation of rodent and human cells [49, 50]. It was observed that only the CREB deficient Tax mutant M47 but not the NF-κB deficient Tax mutant M22, the prototypic mutant in terms of NF-κB deficiency, was able to induce immortalization of T lymphocytes [51, 52]. However, several connections between Tax, NF-κB and tumorigenesis have been described. Importantly, constant activity of NF-κB is a common feature for several ATL-derived cell lines as well as freshly isolated peripheral ATL cells [53]. In line, expression of NF-κB deficient Tax in transgenic mice did not induce ATL-associated skin lesions [54]. Moreover, targeting the NF-κB pathway increased sensitivity of HTLV-1 transformed tumor cells to apoptosis and reduced tumor growth in mice, altogether indicating a decisive role of Tax and NF-κB for HTLV-1 tumorigenicity [55–58].

The role of Tax in inducing NF-κB is well investigated and described. Conversely, the implications of NF-κB on Tax expression, has been less intensively studied. The current study shows a link between NF-κB activity and Tax protein expression. We found that NF-κB deficient Tax mutants show limited protein expression levels which could be rescued upon restoring NF-κB activity in T-cells. Repression of NF-κB in turn led to a significant decrease of Tax protein. Mechanistically, the NF-κB deficient Tax mutant M22 showed lower protein stability than Tax wildtype, but its expression could be enhanced by inhibiting the lysosome and ubiquitinylation. As *Tax* transcript levels remained largely unaffected upon modulation of NF-κB we propose a predominant effect of NF-κB activity on Tax protein level. Taken together, we identified a mechanism that expands our knowledge of the close interplay between NF-κB and Tax-driven transformation.

## Results

### Tax NF-κB mutants are functional but poorly expressed

**Fig. 1:**
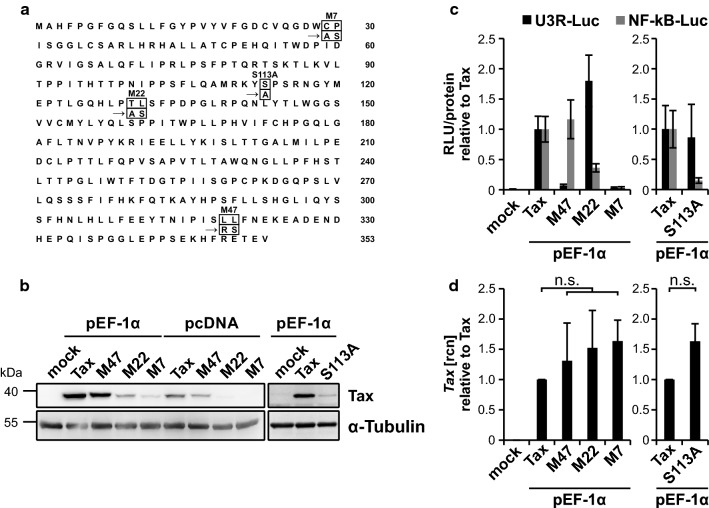
NF-κB deficient Tax mutants are functional but poorly expressed on protein level in Jurkat T-cells. **a** Schematic presentation of the amino acid sequence of Tax protein. Point mutations resulting in the Tax mutant proteins M7 (C29A, P30S), M22 (T130A, L130S), M47 (L319R, L320S) and S113A (S113A) are indicated. **b** Jurkat T-cells were transiently transfected with an empty vector (mock), the Tax-1 expression plasmids Tax (wildtype), M47 (CREB-deficient), M22 (NF-κB-deficient) or M7 (CREB- and NF-κB-deficient), all in the pEF-1α backbone, with the same set of Tax-1 expression plasmids in the pcDNA backbone (50 µg each), or with the pEF-1α based Tax expression plasmids Tax (wildtype) and S113A (30 µg each). Western Blot analysis shows Tax protein and α-Tubulin as a loading control. **c** Jurkat T-cells were co-transfected with an empty vector (mock) or the Tax expression constructs Tax, M47, M22, M7 or S113A, all in the pEF-1α backbone (30 µg each), together with the luciferase reporter vector for the U3R promoter from the HTLV-1 5′ LTR region (U3R-Luc), or a luciferase reporter vector carrying five NF-κB responsive elements (NF-κB-Luc; 20 µg each). Values were normalized on protein content and on luciferase background activity. One representative experiment ± SD is shown. **d** Jurkat T-cells were transfected with an empty vector (mock) or the Tax expression constructs Tax, M47, M22, M7 or S113A, all in the pEF-1α backbone (30 µg each), replenished with 70 µg of an empty vector (pcDNA). *Tax* transcript levels were measured by quantitative PCR (qPCR), and mean relative copy numbers (rcn), normalized on β*-actin* (*ACTB*), of three (M47, M22 and M7) or four (S113A) independent experiments ± SD or SE, respectively, were compared using student’s *t*-test *n.s.* not significant

The HTLV-1 transactivator Tax deregulates and interferes with NF-κB pro-oncogenic signaling [59]. Therefore, protein functions of Tax have been intensively studied focusing not only on its regulatory functions but also on its tumorigenic potential [60]. A valuable model to study functional impacts of Tax is the employment of Tax mutant variants. A panel of Tax mutants, including Tax mutants defective for CREB (M47), NF-κB (M22) or CREB and NF-κB signaling (M7), has frequently been used in terms of HTLV-1 research [51]. Of the 353 aa of Tax, mutation of C29A P30S yielded Tax mutant M7, T130A L131S Tax mutant M22 (originally referred to as Tax M20) and L319R L320S Tax mutant M47 (Fig. 1a). To our surprise, upon expression of the original pc-Tax expression panel in Jurkat T-cells, we noticed that the Tax mutants M22 and M7, which are both defective in NF-κB signaling, expressed much less and close to undetectable limits on Western Blot level compared to Tax wildtype or M47, which is defective in CREB signaling only (Fig. 1b). This effect was predominantly observed in T-cells as protein expression levels of the NF-κB deficient Tax mutants M22 and M7 were only slightly impaired in HEK-293 T cells (Additional file 1. Fig. S1a). We cloned Tax cDNAs from the pcDNA into the pEF-1α vector backbone in order to foster Tax protein expression as expression driven from an EF-1α promoter is assumed to be stronger than from a CMV-driven promoter [61, 62]. Indeed, overall expression levels of the pEF-Tax mutant panel were superior to the pc-Tax constructs when using the same amount of DNA. However, expression patterns with reduced protein amounts of Tax NF-κB mutants M22 and M7 were similar (Fig. 1b). Further, expression analysis of another NF-κB-deficient Tax mutant, S113A [48] (Fig. 1a), confirmed that protein expression of S113A is much less than that of Tax wildtype in Jurkat T-cells. To exclude a general mutational defect, we assured functional properties of the Tax mutants in luciferase-based reporter assays in Jurkat (Fig. 1c) and 293 T cells (Additional file 1: Fig. S1b). For this purpose, we made use of reporter constructs carrying either five NF-κB-responsive elements or the HTLV-1 U3R region [63]. The latter is activated by Tax depending on CREB-signaling [64, 65]. Compared to Tax wildtype, Tax M47 showed a strong decrease in CREB activation but retained NF-κB activity in both vector backbones (Fig. 1c; Additional file 1. Fig. S1b) independent of the cell type tested. In contrast, despite low protein levels, Tax M22 exhibited constant CREB activity but a sharp decline in NF-κB signaling in comparison to Tax wildtype. Correspondingly, S113A showed CREB activity, but was clearly impaired in activating the NF-κB-responsive reporter vectors. As expected, Tax M7 did not show any residual CREB or NF-κB activity (Fig. 1c; Additional file 1. Fig. S1b), indicating an overall robust signaling phenotype. Comparing basal *Tax* mRNA levels of the pEF- and pc-Tax mutant expression panels, no significant discrepancies between the various Tax mutants and Tax wildtype could be observed (Fig. 1d; Additional file 1. Fig. S1c). Thus, different protein expression levels of Tax and NF-κB defective Tax mutants were not due to different amount of *Tax* transcripts, suggesting that Tax’s function to activate NF-κB might be important for proper Tax protein expression in T-cells.

### Recovery of NF-κB activity rescues Tax protein expression

**Fig. 2:**
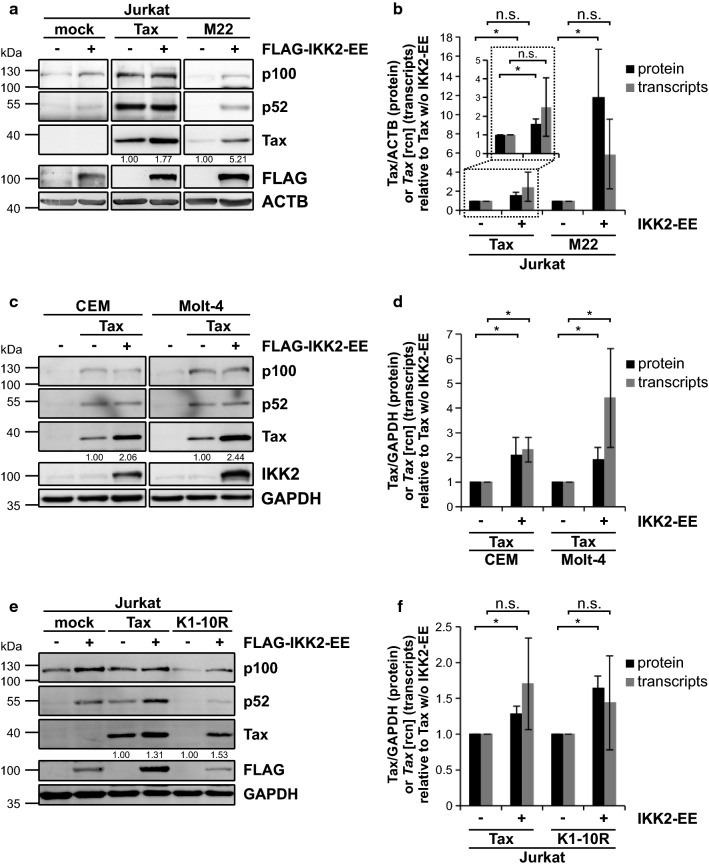
Protein expression of the NF-κB deficient Tax mutants M22 and K1-10R can be rescued by co-expression of IKK2-EE in various T-cell lines. **a**, **b**, **e**, **f** Jurkat T-cells or **c**, **d** CCRF-CEM and Molt-4 T-cells were transfected with the Tax wildtype expression plasmid (**a**–**d**) pEF-Tax1 (Tax, 30 µg) or (**e**, **f**) pSG5-Tax (Tax, 5 µg) and the NF-κB-deficient Tax mutant (**a**, **b**) pEF-M22 (M22, 30 µg) or (**e**, **f**) pSG5-Tax-K1-10R (K1-10R, 15 µg) together with the IKK2 constitutive active mutant pcFLAG-IKK2-EE (FLAG-IKK2-EE, 40 µg) or the empty vector (pcDNA). All samples were replenished to 100 µg with the respective empty vector DNA (pcDNA, pSG5). **a**, **c**, **e** Immunoblot shows Tax, NF-κB2 p100/p52, FLAG or IKK2 and ACTB or GAPDH as controls. Numbers depict a representative densitometric analysis of Tax protein expression levels normalized on ACTB or GAPDH and Tax expression w/o IKK2-EE. **b**, **d**, **f** Densitometric analysis of Tax protein expression relative to ACTB or GAPDH, normalized on Tax expression w/o IKK2-EE, of at least three independent experiments was performed (protein, black bars). Values ± SD are depicted. Corresponding *Tax* transcript levels were measured by quantitative PCR (qPCR; transcripts, grey bars). Mean relative copy numbers (rcn), normalized on β*-actin* (*ACTB*) and on Tax w/o IKK2-EE, of three independent experiments ± SD were compared using student’s *t*-test (*p < 0.05; *n.s.* not significant). The inset in (**b**) shows an enlargement of Tax protein and *Tax* transcript levels derived from Tax wildtype transfected Jurkat T-cells

The interplay between Tax and components of the NF-κB signaling pathway is multifaceted and highly complex [66, 67]. In any case, Tax-1 of HTLV-1 is capable of inducing not only canonical but also non-canonical or alternative NF-κB signaling [68–70]. In Jurkat T-cells, this effect is observed with an antibody targeting NF-κB2 p100/p52, which recognizes at the same time p100 and p52 as a readout for canonical and non-canonical NF-κB activity, respectively. Expression of Tax resulted in robust p100 and p52 activity whereas Tax M22 alone showed weak protein levels not only for M22 itself but also for p100 and p52 (Fig. 2a). In order to boost endogenous NF-κB activity in transfected T-cells and to test whether this also enhances Tax protein expression, we made use of the IKK2 variant IKK2-EE, which mimics constant phosphorylation in its activation loop and, thus, continuously activates canonical NF-κB signaling [71]. Expression of IKK2-EE alone resulted in slight induction of p100. Co-transfection of Tax with IKK2-EE resulted in an increase of p100 and p52 levels, especially in the context of M22. Strikingly, not only Tax but particularly M22 protein levels vastly increased in the presence of IKK2-EE (Fig. 2a). As immunoblots were performed using fluorescently labeled antibodies, densitometric analysis could be applied, which revealed a significant increase of Tax and Tax M22 protein levels in the presence of IKK2-EE (Fig. 2b). This effect of NF-kB activity on Tax protein did not apply to *Tax* transcripts as corresponding mRNA levels only tended to increase and varied (Fig. 2b). To exclude that the increase of Tax protein upon NF-κB stimulation was due to a Jurkat T-cell artifact, we repeated the experiment in CCRF-CEM and Molt-4 CD4^+^ T-cell lines. Expression of Tax (Fig. 2c) and of the Tax mutant M22 (Additional file 2. Fig. S2a) yielded a robust induction not only of Tax protein itself but also of p100 and p52 in CEM as well as in Molt-4 T-cells. As in Jurkat T-cells, co-expression of IKK2-EE led to a significant increase of Tax and Tax M22 protein also in CEM and Molt-4 T-cells as revealed by densitometric analysis (Fig. 2c, d; Additional file 2. Fig. S2b). However, in contrast to Jurkat T-cells, corresponding *Tax* transcript levels were significantly elevated in CEM and Molt-4 T-cells in the presence of IKK2-EE (Fig. 2d; Additional file 2. Fig. S2b). To prove a general boost of Tax protein by increased NF-κB activity, we additionally analyzed the Tax mutant K1-10R, which was generated by replacing the ten lysine residues of Tax-1 with arginine [72]. This Tax variant is no longer ubiquitinylated and, thus, no longer confers NF-κB activity. Like the other NF-κB deficient Tax mutant M22, expression of Tax K1-10R in Jurkat T-cells showed low protein levels of Tax K1-10R itself as well as p100 and p52, compared to Tax wildtype (Fig. 2e), thus, strengthening our hypothesis that Tax’s capacity to activate NF-κB is important for Tax protein stabilization. Expression of IKK2-EE not only alone but also together with Tax and Tax K1-10R resulted in an increase of p100 and p52 as well as Tax and Tax K1-10R protein (Fig. 2e). The significant increase of Tax protein in the presence of IKK2-EE, as proven by densitometric analysis, did not exhibit on mRNA level as *Tax* transcripts only slightly tended to increase (Fig. 2f). Therefore, protein stimulatory effects of IKK2-EE on Tax are not in general reproduceable on transcript level. Together, these data show that activation of NF-κB by IKK2-EE enhances expression of Tax and the Tax mutants M22 and K1-10R in T-cells.

### Tax protein levels are elevated independent of the promoter

**Fig. 3:**
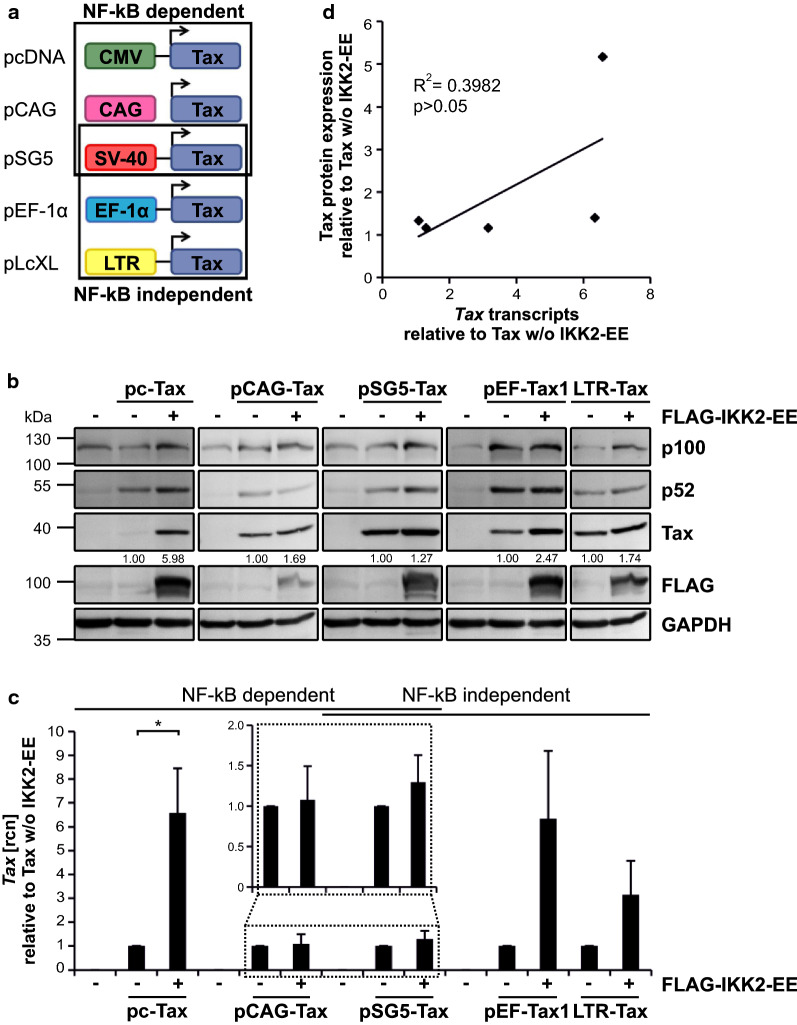
Tax protein levels are elevated independent of the promoter. **a** Schematic representation of the Tax expression constructs and their promoters used in (**b**–**d**). **b–d** Jurkat T-cells were transiently transfected with the Tax expression constructs pc-Tax, pCAG-FLAG-Tax (pCAG-Tax), pSG5-Tax, pEF-Tax1 or pLcXL (LTR-Tax; 30 µg each) and the IKK2 constitutive active mutant pcFLAG-IKK2-EE (FLAG-IKK2-EE) or the empty vector (pcDNA; 40 µg each). All samples were replenished to 100 µg with empty vector pcDNA. **b** Western Blot shows Tax, NF-κB2 p100/p52, FLAG and GAPDH as controls. Numbers depict a representative densitometric analysis of Tax protein expression levels normalized on GAPDH and Tax expression w/o IKK2-EE. **c** *Tax* transcript levels were measured by quantitative PCR (qPCR). Mean relative copy numbers (rcn), normalized on β*-actin* (*ACTB*) and on Tax w/o IKK2-EE, of four independent experiments ± SE are shown and were compared using student’s *t*-test (*p < 0.05). The inset shows an enlargement of *Tax* transcript values derived from pCAG-Tax and pSG5-Tax transfected Jurkat T-cells. **d** Correlation between Tax protein values, derived from four independent experiments as depicted in (**b**), and their respective transcript levels as depicted in (**c**) was calculated using Pearson’s correlation (R^2^ = 0.3982; *p* > 0.05)

Plasmid-based transient expression of transgenes is dependent on various factors. Thereby, a decisive role is not only played by the cell type but also by the promoter which is applied to drive ectopic gene expression [62]. As several commonly used constitutive promoters additionally bear elements that are regulated by NF-κB signals, we aimed to exclude promoter-dependent effects of IKK2-EE on Tax protein expression. Therefore, we made use of a Tax expression construct panel, consisting of pc-Tax [73] (CMV promoter, NF-κB dependent), pCAG-FLAG-Tax [74] (chimeric promoter consisting of CMV immediate early enhancer, chicken β-actin promoter and rabbit β-globin intron, NF-κB dependent), pSG5-Tax [75] (SV40 promoter, potentially NF-κB dependent), pEF-Tax1 (EF-1α promoter, NF-κB independent), and pLcXL [76] (LTR-Tax; HTLV-1 LTR as promoter, NF-κB independent) [51, 77–81] (Fig. 3a). Transient expression of pc-Tax, pCAG-FLAG-Tax, pSG5-Tax, pEF-Tax1 and LTR-Tax in Jurkat T-cells resulted in robust activation of p100 and p52 as well as varying Tax expression levels, ranging from low to very strong (Fig. 3b). Co-expression of the IKK2 constitutive active mutant, IKK2-EE, led to an increase of Tax protein irrespective of the promoter that was used to drive Tax expression, as indicated by densitometric analysis (Fig. 3b). Contrary, corresponding mRNA levels revealed varying effects. The pc-Tax expression construct showed significantly elevated amounts of *Tax* transcripts in the presence of IKK2-EE whereas *Tax* mRNA produced by any other Tax expression construct was only moderately (pEF), slightly (LTR) or not affected at all (pCAG, pSG5) by IKK2-EE (Fig. 3c). Correlating the increase of Tax protein upon co-expression with IKK2-EE with its respective induction of *Tax* transcripts, it is conceivable that elevated amounts of Tax protein did not coincide with their respective *Tax* mRNA levels (*p* > 0.05; Fig. 3d). Thus, a promoter-independent increase of Tax protein upon activation of NF-κB potentially occurred irrespective of *Tax* mRNA.

### Tax expression is fostered by induction of canonical NF-κB signaling with TPA/ ionomycin, but not with TNF-α

NF-κB activity is tightly regulated in order to modulate expression of immunomodulatory genes. In T cells, two major stimuli can induce activation of the NF-κB pathway, (1) ligation of the Tumor Necrosis Factor Receptor 1 (TNFR1) by its ligand TNF-α, and (2) triggering of the T cell receptor (TCR) by an MHC-II-bound antigen peptide [82, 83]. In order to further elucidate the mode of action of NF-κB activation on Tax protein expression, we induced NF-κB signaling by the cytokine TNF-α, which activates canonical NF-κB signaling in a broader range at the very top of the signaling cascade [84]. Treatment of Jurkat T-cells with TNF-α alone led to a rapid induction of p100 protein as soon as 15 min after induction, indicating a working experimental setting (Additional file 3. Fig. S3a). Expression of Tax or Tax M22 alone led to robust or weak, respectively, induction of Tax, p100 and p52 protein as observed earlier (Additional file 3. Fig. S3a). Further stimulation of NF-κB in Tax or Tax M22 expressing cells with TNF-α for 15 min, 60 min or 24 h led to a more pronounced increase of p100, however, protein levels of Tax and Tax M22 remained either unaltered (15 and 60 min TNF-α) or even slightly decreased (24 h TNF-α), as verified by densitometric analysis (Additional file 3. Fig. S3a, b). In accordance with protein levels, *Tax* mRNA was largely unaffected by treatment of cells with TNF-α except at 24 h post treatment where also *Tax* transcripts declined (Additional file 3. Fig. S3c). Thus, in contrast to IKK2-EE, stimulation of NF-κB by the cytokine TNF-α did not show an elevating impact on Tax protein level, suggesting that TNF-α treatment is either too weak, or that components located further downstream in the NF-κB signaling cascade contribute to enhance Tax protein levels.

**Fig. 4:**
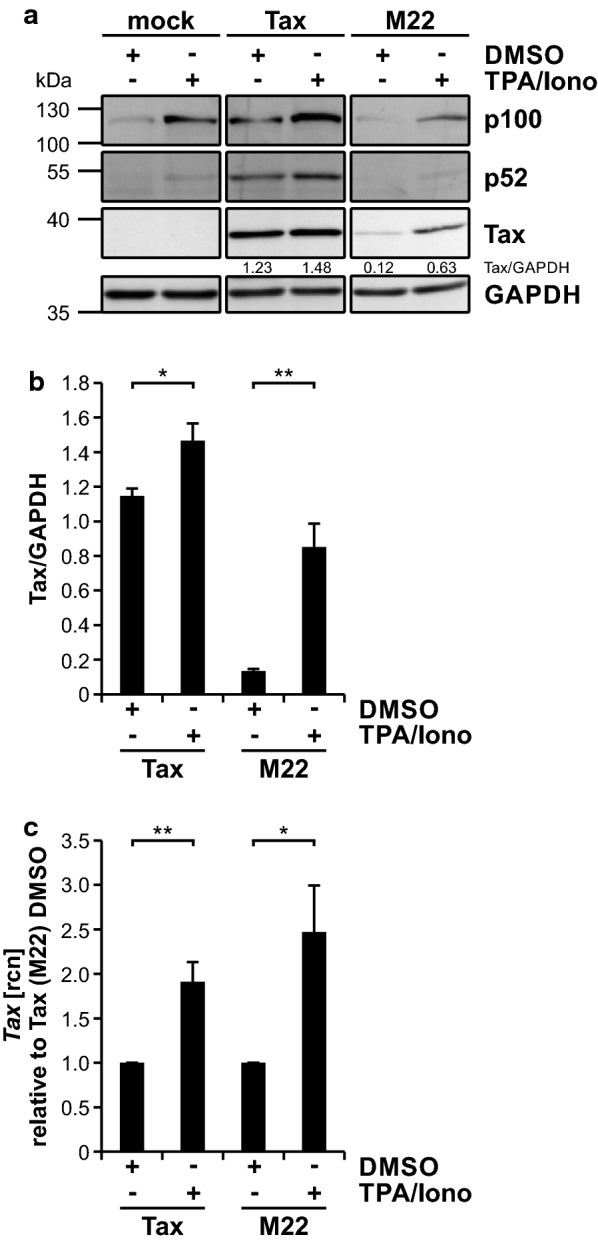
Expression of Tax and the NF-κB deficient Tax mutant M22 is fostered by induction of NF-κB with TPA/ ionomycin. **a**–**c** Jurkat T-cells were transiently transfected with EF1-α-driven Tax expression plasmids Tax (wildtype; 20 µg), M22 (NF-κB-deficient) or mock (100 µg each), replenished, where necessary, to 100 µg with the empty vector. At 24 h after transfection, cells were treated with 20 nM 12-O-Tetradecanoylphorbol-13-Acetate (TPA) and 1 µM ionomycin or the solvent control DMSO for another 24 h. **a** Immunoblot shows Tax, NF-κB2 p100/p52 and GAPDH. Numbers depict a representative densitometric analysis of Tax protein expression levels normalized on GAPDH. **b** Densitometric analysis of Tax protein expression relative to GAPDH, of four independent experiments was performed. Values ± SE are depicted and were compared using student’s *t*-test (*p < 0.05; **p < 0.01). **c** mRNA of *Tax* was measured by quantitative PCR (qPCR). Mean relative copy numbers (rcn), normalized on β*-actin* (*ACTB*) and on values derived from the DMSO solvent control, of four independent experiments ± SE are shown and were compared using student’s *t*-test (*p < 0.05; **p < 0.01)

To mimic and induce antigen-receptor-stimulated NF-κB activation much stronger than by TNF-α, Jurkat T-cells were treated with 12-O-tetradecanoylphorbol-13-acetate (TPA; Phorbol 12-myristate 13-acetate (PMA)) and ionomycin [85]. TPA is considered to activate the IKK complex through activation of protein kinase C. Stimulation of Jurkat T-cells with TPA (20 nM) plus ionomycin (1 µM) for 24 h led to induction of p100 expression indicating activity of canonical NF-κB signaling (Fig. 4a). Interestingly, expression of Tax wildtype, and of M22, significantly increased upon treatment with TPA/ ionomycin both on protein (Fig. 4a, b) and on transcript level (Fig. 4c). Together, these data show that TPA/ ionomycin induces Tax wildtype and restores M22 expression in T-cells, presumably by induction of canonical NF-κB signaling.

### Repression of NF-κB leads to reduced amounts of Tax protein

**Fig. 5:**
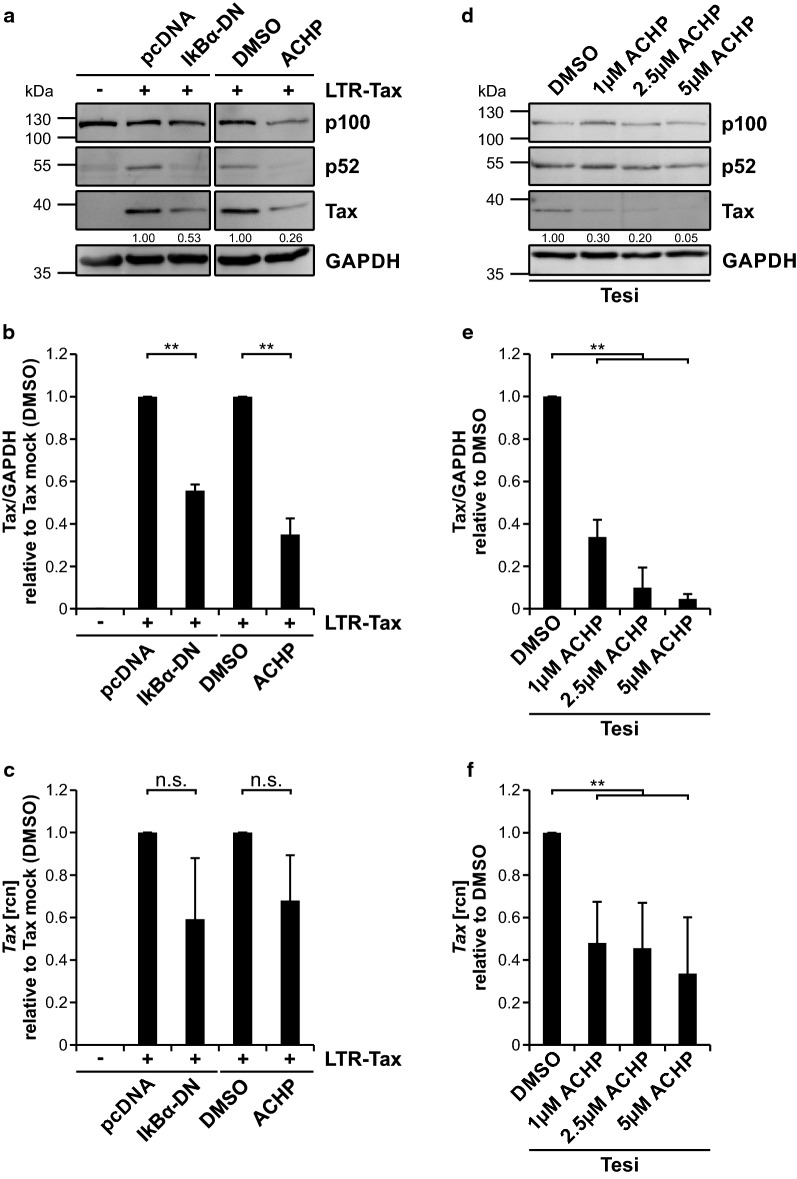
Tax protein is diminished predominantly over *Tax* transcript levels upon repression of NF-κB activity. **a**–**c** Jurkat T-cells were transfected with the Tax expression plasmid pLcXL (LTR-Tax; 20 µg) alone (mock) or together with a dominant negative inhibitor of κB pIκBα-DN (IκBα-DN; 5 µg), replenished to 100 µg DNA with the empty vector pcDNA. At 4 h after transfection, transfected cells were treated, where indicated, for 44 h with 2.5 µM of the IKK2 inhibitor 2-Amino-6-(2-(cyclopropylmethoxy)-6-hydroxyphenyl)-4-(4-piperidinyl)-3-pyridinecarbonitrile (ACHP) or the solvent control DMSO. **a** Western Blot shows Tax, NF-κB2 p100/p52 and GAPDH. Numbers indicate a representative densitometric analysis of Tax protein expression levels normalized on GAPDH and Tax expression w/o IκBα-DN or the solvent control DMSO. The blot was cut due to technical reasons. **b** Densitometric analysis of Tax protein, normalized on GAPDH and Tax w/o IκBα-DN or the solvent control DMSO, of three independent experiments was performed. Values ± SD are depicted and were compared using student’s *t*-test (**p < 0.01). **c** *Tax* transcript levels were measured by quantitative PCR (qPCR). Mean relative copy numbers (rcn), normalized on β*-actin* (*ACTB*) and Tax w/o IκBα-DN or the solvent control DMSO, of four independent experiments ± SE are shown and were compared using student’s *t*-test (n.s., not significant). **d–f** Tax-transformed Tesi cells were treated with 1 µM, 2.5 µM or 5 µM of the IKK2 inhibitor ACHP or the solvent control DMSO. **d** Western Blot shows Tax, NF-κB2 p100/p52 and GAPDH. Numbers indicate a representative densitometric analysis of Tax protein expression levels normalized on GAPDH and Tax expression in DMSO treated cells. **e** Densitometric analysis of Tax protein, normalized on GAPDH and Tax in DMSO treated cells, of three independent experiments was performed. Values ± SD are depicted and were compared using student’s *t*-test (**p < 0.01). **f** *Tax* transcript levels were measured by quantitative PCR (qPCR). Mean relative copy numbers (rcn), normalized on β*-actin* (*ACTB*) and values derived from DMSO treated cells, of three independent experiments ± SD are shown and were compared using student’s *t*-test (**p < 0.01)

Unchecked and excessive induction of NF-κB signaling is not only a benchmark of Tax-mediated cellular deregulation but a general hallmark during the development of various types of cancers [86, 87]. Therefore, much effort has been put into the identification of mechanisms and molecules in order to repress deregulated NF-κB signaling. In order to analyze the effect of repression of endogenous NF-κB signaling on Tax protein expression, we took two approaches: making use of a dominant-negative inhibitor of κB, IκBα-DN, which competes with endogenous IκBα [88], and the chemical compound 2-Amino-6-(2-(cyclopropylmethoxy)-6-hydroxyphenyl)-4-(4-piperidinyl)-3-pyridinecarbonitrile (ACHP), an inhibitor of the IKK subunit IKK2 [89]. As expected, Tax expressing Jurkat T-cells robustly exhibited p100 and p52 activity compared to untransfected cells (Fig. 5a). On the other hand, repression of NF-κB signaling either by IκBα-DN or by ACHP showed a sharp decline in p100 and p52 protein level which subsequently led to a significant reduction of Tax protein in comparison to mock co-transfected or DMSO treated cells (Fig. 5a, b). Repression of NF-κB by IκBα-DN or ACHP exerted a more pronounced effect on Tax protein than transcript expression as *Tax* mRNA levels were only mildly affected and did not show a significant impairment (Fig. 5c). To discover whether Tax is also affected by repression of NF-κB in context of a Tax-immortalized T-cell line, we treated Tesi cells with increasing amounts of ACHP. Tesi cells are derived from primary human T-cells immortalized by an expression cassette for Tax, which was transduced with a rhadinoviral vector featuring tetracycline-repressible Tax expression [90]. Western blot analysis and corresponding densitometric analysis revealed that increasing, but non-toxic concentrations of the IKK2-inhibtior ACHP [91] lead to a significant and dose-dependent decrease of Tax protein expression reaching up to 95% of protein repression at the highest ACHP concentration used (Fig. 5d, e). ACHP also impaired *Tax* transcripts significantly by 60–70% (Fig. 5f), but to a lesser extent than Tax protein expression (Fig. 5e). Conclusively, repression of endogenous NF-κB signaling dominantly reduced Tax protein over *Tax* transcripts. Together with our previous data, this supports the notion that Tax protein is stabilized by NF-κB activity.

### Protein stability of Tax M22 is limited compared to Tax wildtype

**Fig. 6:**
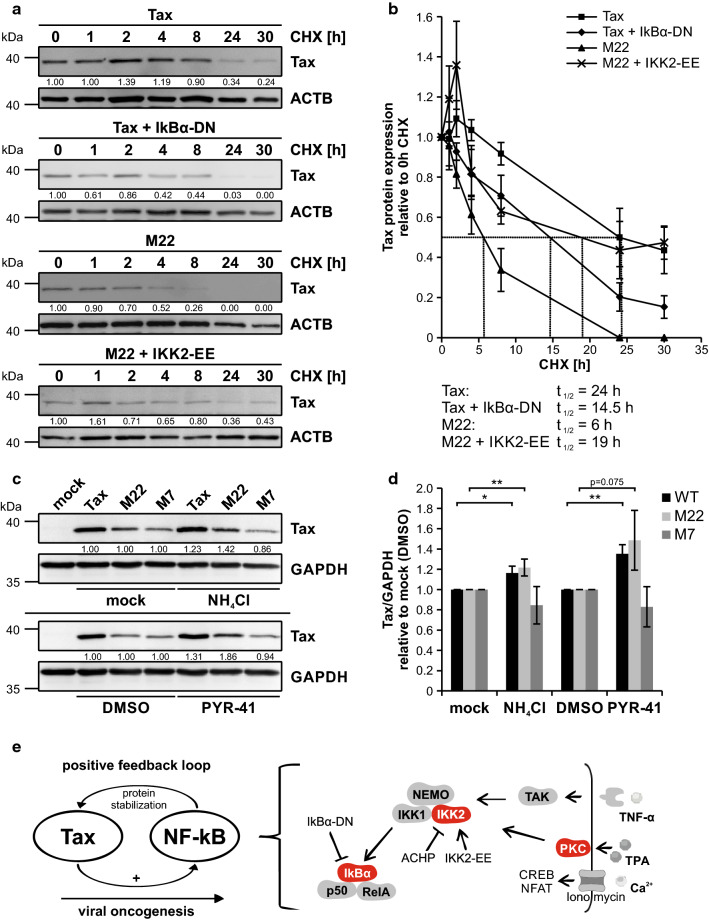
NF-κB activity is important for protein stability. **a**, **b** Jurkat T-cells were transfected with EF1-α-driven Tax expression plasmids Tax (wildtype) or Tax mutant M22 (NF-κB-deficient; 40 µg each), Tax together with pIκBα-DN (10 µg), a dominant negative inhibitor of κB (IκBα-DN), or M22 together with pc-FLAG-IKK2-EE (40 µg), a constitutive active variant of IKK2 (IKK2-EE). All samples were replenished to 100 µg DNA with the empty vector pcDNA. At 24 h after transfection, cells were treated for the indicated periods of time with 50 µg/ml cycloheximide (CHX). **a** Immunoblot shows Tax and ACTB. Numbers indicate representative densitometric analysis of Tax protein expression levels normalized on Tax expression at 0 h CHX. **b** Densitometric values derived from (**a**), relative to 0 h CHX, are depicted for Tax (closed squares), Tax together with IκBα-DN (closed diamonds), M22 (closed triangles) and M22 together with IKK2-EE (black crosses). Each experiment was independently performed in quadruplicate and values ± SE are shown. Protein half-life of Tax or M22, respectively, were empirically determined as indicated. **c**, **d** Jurkat T-cells were transfected with Tax-1 expression plasmids Tax (wildtype; 40 µg), M22 (NF-κB-deficient) or M7 (CREB- and NF-κB-deficient; 100 µg each), all in the pEF-1α backbone, replenished, where necessary, to 100 µg DNA with empty vector DNA. At 24 h after transfection, transfected cells were treated, where indicated, for 24 h with the water-dissolved lysosome inhibitor Ammonium chloride (NH_4_Cl, 20 mM), the ubiquitin-activating enzyme E1 inhibitor 4[4-nitro-furan-2-ylmethylene)-3,5-dioxo-pyrazolidin-1-yl]-benzoic acid ethyl ester (PYR-41; 15 µM) or the solvent control DMSO. **c** Western Blot shows Tax and GAPDH. Numbers indicate a representative densitometric analysis of Tax protein expression levels normalized on GAPDH and the respective Tax expression in mock or DMSO treated cells. **d** Densitometric analysis of Tax protein, normalized on GAPDH and the respective Tax expression in mock or DMSO treated cells, of four independent experiments was performed. Values ± SE are depicted and were compared using student’s *t*-test (*p < 0.05; **p < 0.01). **e** Schematic working model of a potential positive feedback loop between Tax and NF-κB activity. Components of the NF-κB signaling cascade with an experimentally shown impact on Tax protein expression are colored in red

Data generated in this study so far indicated a predominant role of NF-κB in Tax protein expression. In the next step, we aimed to find out mechanistic implications of NF-κB signaling on Tax protein. Therefore, in order to ascertain potential differences in protein turnover between Tax wildtype and M22, we performed cycloheximide (CHX) chase assays, a well-established method to determine protein stability [92]. Transient transfection of Tax in Jurkat T-cells showed robust signals for Tax protein on Western Blot level. Incubation of Tax expressing T-cells for increasing periods of time with CHX showed constant reduction of Tax protein, almost vanishing 30 h after addition of CHX (Fig. 6a). Integrating densitometric data of Tax Western Blots into numbers, the half-life of Tax protein was calculated to be approximately 24 h (Fig. 6b, closed squares). In contrast, Tax M22 already showed lower amounts of basal Tax protein expression, as revealed by densitometric analysis (Fig. 6a, Additional file 4. Fig. 4). Strikingly, expression levels of Tax M22 were rapidly diminished upon treatment with CHX, exhibiting a much faster decay than Tax wildtype as protein half-life of Tax M22 was only around 6 h (Fig. 6b, closed triangles). According to previous findings, co-expression of Tax and IκBα-DN yielded reduced amounts of Tax protein compared to expression of Tax alone (Fig. 6a, Additional file 4. Fig. S4). Interestingly, repression of NF-κB by IκBα-DN increased Tax protein turnover rate and vastly reduced its protein half-life to 14.5 h (Fig. 6b, closed diamonds), supporting the idea that Tax’s capability to induce NF-κB is critical for protein stabilization of Tax. On the other hand, IKK2-EE enhanced the protein half-life of M22 from 6 h (Fig. 6b, closed triangles) to approximately 19 h (Fig. 6b, black crosses). Thus, these findings indicate that protein stability of Tax M22 is strongly reduced compared to Tax wildtype, but can be stabilized by activating NF-κB signaling.

To shed light on the potential mechanism of Tax protein stabilization, we co-incubated Jurkat T-cells after transfection of Tax wildtype, M22 or M7 with NH_4_Cl, a broad inhibitor of lysosomal degradation, and with 4[4-(5-nitro-furan-2-ylmethylene)-3,5-dioxo-pyrazolidin-1-yl]-benzoic acid ethyl ester (PYR-41), which blocks the ubiquitin-activating enzyme E1 [93]. Protein expression of both Tax wildtype and M22 increased upon treatment with NH_4_Cl and PYR-41 compared to mock or solvent (DMSO) treated controls (Fig. 6c, d). Strikingly, protein expression of M7, which is defective in both NF-κB and CREB signaling, did not increase upon inhibitor treatment (Fig. 6c, d), hinting at a role for Tax’s intrinsic CREB-activity in protein destabilization by lysosomal degradation and by ubiquitinylation.

Taken together, our work shows that Tax does not only induce NF-κB, but that Tax protein expression vice versa depends on NF-κB activity, suggesting a positive feedback loop between Tax and NF-κB (Fig. 6e, left side). Our current model proposes that NF-κB activity contributes to Tax protein stability as experimental modulation of NF-κB activity resulted in altered protein levels of Tax, potentially due to preventing lysosomal and/ or ubiquitin-dependent degradation of Tax (Fig. 6e, right side). At the same time, *Tax* transcript levels were much less affected, putting forward the notion that the positive feedback loop exerts its function predominantly on protein level.

## Discussion

Tax, the HTLV-1 oncoprotein, is an important factor of pathogenicity, and it is the causative agent for initiating transformation of infected CD4^+^ T-cells leading to HTLV-1 associated diseases like ATL/L. Constitutive activation of NF-κB, even independent at a certain point of Tax expression due to epigenetic silencing of the HTLV-1 promoter, is fundamental for aberrant growth and induction of leukemia [94–96]. In previous studies, we and others identified a plethora of NF-κB-dependent Tax target genes that might contribute to HTLV-1-driven cancer [10, 91, 97–100]. In this study, we found that Tax protein expression is affected by NF-κB activity, suggesting a positive feedback loop between Tax and NF-κB in T-cells.

Unexpectedly, we could show that protein expression levels of the NF-κB deficient Tax mutants M22 and K1-10R were vastly diminished in T-cells despite different types of mutations. While M22 carries two point mutations (T130A L131S [51]), the Tax mutant K1-10R was generated by replacing the ten lysine residues to arginine [72, 101]. In its native state, K63-linked ubiquitinylated Tax recruits the IKK-γ (NEMO) regulatory subunit of the IKK complex into the centrosome/ Golgi-associated structures, followed by activating the IKK complex, and thus, NF-κB [102, 103]. The Tax mutant M22 is no more able to physically bind to NEMO, is impaired in dimerization and therefore is incapable to induce NF-κB [32, 104]. Tax K1-10R lacks attachment points for classical ubiquitinylation which is indispensable for Tax mediated activation of NF-κB [72, 101, 105]. Additionally, as for M22, Tax K1-10R is no more able to physically interact with NEMO [106]. Therefore, Tax ubiquitinylation is thought to mediate binding to NEMO. The finding that fusion of M22 to NEMO restores the capacity of M22 to induce NF-κB further stresses the importance of a Tax-NEMO interaction for induction of NF-κB activity [107]. Taken together, diminished expression of the mutants M22 and K1-10R is not due to a feature of a certain mutation but rather due to a functional defect in NF-κB activation.

Activation of canonical as well as non-canonical NF-κB signaling depends on the activation of the IKK complex. IKK2 is specifically involved in canonical NF-κB signaling, forming an IKK1-IKK2 heterodimer. In contrast, the regulatory subunit of the IKK complex in non-canonical NF-κB signaling consists of an IKK1 homodimer [108]. Hence, we made use of an IKK2 mutant, which mimics constant phosphorylation in its activation loop by site-specific mutation (S177E, S181E), IKK2-EE [71]. This mutant exclusively induces canonical NF-κB signaling, however, we noticed basal proteolytic degradation of p100 to p52 upon expression of IKK2-EE alone. This potentially unexpected crosstalk between the two NF-κB pathways on IKK2 level might be explained by the fact that a variety of proteins serve as IKK2 substrates, including p105 [109]. Interestingly, co-expression of Tax or Tax M22 with IKK2-EE elevated protein levels significantly, not only of Tax wildtype but also of the NF-κB deficient Tax mutants M22 and K1-10R in Jurkat T-cells. Activation of NF-κB by Tax therefore seems to be dispensable for Tax protein stabilization and can be substituted by heterologous NF-κB activation. Moreover, IKK2-EE was able to elevate Tax and Tax M22 protein levels also in CCRF-CEM and Molt-4 CD4^+^ T-cell lines. Of note, expression defects of NF-κB deficient Tax mutants were not visible pronouncedly in the epithelial cell line 293 T, which lacks T-cell specific co-factors, indicating a cell type specific effect [110]. As many studies focusing on Tax protein functions were conducted in 293 T or other adherent cell types like HeLa, but HTLV-1 naturally infects and persists in CD4^+^ T-cells, these findings should be carefully considered. Depending on the T-cell line, corresponding *Tax* transcript levels were alternating elevated in the presence of IKK2-EE. On the one hand, this hints at the fact that NF-κB signaling might be differentially modulated between T-cell lines, presumably via alternative phosphorylation events [111]. On the other hand, the dominant effect of NF-κB activation on Tax expression might be exerted on protein level, irrespective of the induction of *Tax* transcripts. Intriguingly, stimulation of NF-κB by TNF-α was not able to elevate Tax protein levels while TPA/ ionomycin treatment led to an increase of Tax wildtype and, even more pronounced, of M22 expression. TNF-α activates canonical NF-κB signaling by binding to the TNF receptor (TNF-R) and subsequent phosphorylation of TAK1 and activation of the IKK complex [112]. TPA/ ionomycin are potent inducers of NF-κB and cytokine signaling, and TPA activates the IKK complex more downstream through protein kinase C (PKC), while TNF-α-mediated NF-κB activation is independent of PKC in Jurkat T-cells [113]. However, earlier work has shown that TPA/ ionomycin is also able to activate nuclear factor of activated T-cells (NFAT) signaling together with Tax and M22 [114], thus crosstalk of other signaling pathways than NF-κB cannot be excluded. Hence, modulation of NF-κB by IKK2-EE represents a more direct mode of pathway modulation by circumventing extracellular receptors and other signaling pathways. Therefore, data indicate that components downstream of receptor signaling mediate alteration of Tax protein expression. This assumption is strengthened by the notion that Tax activates NF-κB downstream of TNF-α in the NF-κB signaling cascade [115]. Of note, the finding that TNF-α alone, similar to M22 but in contrast to Tax, was not able to target NEMO to the centrosome [102] indicates that Tax-mediated NEMO interaction and re-localization essentially contributes to the regulation of Tax protein expression. Future studies should narrow down whether the binding of Tax to NEMO and the capacity of Tax to relocalize IKK to Golgi-associated structures or the centrosome [102, 103] are critical for modulating Tax protein expression. Aiming into this direction, we performed experiments repressing NF-κB activity. For instance, irrespective of the mode of stimulation, activation of NF-κB depends on the degradation of IκB proteins, which keep Rel proteins in an inactive state in the cytoplasm. Phosphorylation of IκB in its native state is a prerequisite for its degradation. Therefore, a dominant-negative mutant of IκBα (IκBα-DN), engineered to be unable to be phosphorylated, is resistant to degradation and, thus, constantly blocks NF-κB activity [88, 116]. Upon repression of canonical NF-κB making use of IκBα-DN and/ or ACHP, a selective inhibitor of IKK2 [89], we significantly repressed Tax protein levels not only in Tax-transfected but also in Tax-immortalized cells, excluding transfection artifacts. The finding that *Tax* mRNA remained either largely unaffected in Tax-transfected or much less affected in Tax-transformed cells further stresses the idea of a dominant protein modulatory mechanism.

Aiming for the underlying mechanism, we performed CHX chase assays to determine Tax protein stability under NF-κB modulatory conditions. We could show that the turnover rate of Tax M22 protein is much higher compared to Tax wildtype which is in line with previous findings that M22 is no longer ubiquitinylated [106]. The hypothesis that NF-κB activity predominantly affects Tax protein is further stressed by the findings that inhibition of NF-κB seemed to destabilize Tax protein, while IKK2-EE prolonged the half-life of M22. Future work should determine the detailed cellular pathways which are responsible for Tax protein (de)stabilization. Our work identified a crucial role of the lysosome since inhibition of lysosomal degradation enhanced Tax protein expression and strongly reconstituted M22 expression. It is also likely that ubiquitinylation, a major pathway interfering with Tax protein expression, is involved in NF-κB-mediated protein stabilization of Tax. This assumption is supported by the fact that chemical inhibition of the ubiquitin-activating enzyme E1 led to an increase of Tax and M22, although it cannot be excluded that this effect is indirect by attenuating cytokine-mediated NF-κB activation [93]. Tax utilizes RNF8 and Ubc13, ubiquitin E3 ligase or E2 conjugating enzymes, respectively, to activate NF-κB activity [39, 43]. Furthermore, p62 has been identified as a new ubiquitin-dependent modulator of Tax activity on NF-κB [46]. Importantly, Tax has been shown to be ubiquitinylated not only upon ectopic expression in 293 T cells but also in transfected T-cells and in chronically infected T-cell lines [72, 106, 117]. It was also demonstrated that although ubiquitinylated Tax, in contrast to Tax K1-10R and M22, binds to the proteasome, proteasomal inhibition did not foster Tax protein expression [72, 117, 118]. This finding presumably rules out proteasomal degradation as the driving mechanism in modulating Tax protein expression in the present study. The insight that activation of NF-κB by IKK2-EE could rescue protein levels of the lysine-depleted Tax mutant K1-10R would contradict the hypothesis that ubiquitinylation stabilizes Tax protein. However, apart from canonical K-linked ubiquitinylation, non-canonical N-terminal, C-, S- or T-linked ubiquitinylation has been described [119]. Although it has been clearly demonstrated that Tax K1-10R is not ubiquitinylated in 293 T cells [72, 106], the possibility that Tax or Tax K1-10R could be ubiquitinylated in T-cells by non-canonical ubiquitinylation should not be ruled out. Moreover, a crosstalk between ubiquitinylation and SUMOylation might be conceivable as Tax M22 not only shows defective ubiquitinylation but also SUMOylation [106]. The findings that Tax SUMOylation is dispensable for induction of NF-κB activity [120] but critical for the formation of RelA-enriched Tax nuclear bodies [106] indicate that modulation of Tax protein expression by the SUMO pathway occurs via non-exclusive NF-κB signaling events.

## Conclusions

Overall, our findings suggest a novel connection between Tax and NF-κB activity. Tax protein itself seems to hijack NF-κB signaling in order to augment its protein stability. The observation of a positive feedback loop between viral oncoproteins and cellular factors seems to occur rarely as it was, until now, only described for the Epstein-Barr virus (EBV) Latent Membrane Protein 1 (LMP1) and Signal transducer and activator of transcription 3 (STAT3) [121]. Therefore, elucidating the biological impact of this regulation will be relevant in order to gain more knowledge on the interplay between Tax and NF-κB signaling. Interfering more precisely with NF-κB signaling might be helpful to enlarge treatment options in early stages of Tax-induced oncogenesis.

## Methods

### Cell lines

The CD4^+^ T-cell lines Jurkat [122], CCRF-CEM [123], and Molt-4 [124] were cultivated in RPMI 1640 (45%; GIBCO, Life Technologies, Darmstadt, Germany) and Panserin 401 medium (45%; PAN-Biotech, Aidenbach, Germany), supplemented with 10% fetal calf serum (FCS; Sigma Aldrich, Darmstadt, Germany), L-glutamine (0.35 g/l) and penicillin/streptomycin (Pen/Strep; 0.12 g/l each). HEK-293 T cells were cultured in DMEM (GIBCO, Life Technologies) containing 10% FCS, L-glutamine and Pen/Strep. Tesi cells are primary human T-cells immortalized by an expression cassette for Tax, which was transduced with a rhadinoviral vector featuring tetracycline-repressible Tax expression [90]. Tesi cells were cultivated in RPMI 1640 (40%) and Panserin 401 medium (40%), supplemented with 20% FCS, L-glutamine, Pen/Strep and 40 U/ml interleukin-2 (IL-2; Roche Diagnostics, Mannheim, Germany).

### Plasmids and transfection

#### Plasmids

The following plasmids were used for transient transfection experiments: pcDNA3.1, pEF-1α (both Life Technologies), pSG5 (Agilent Technologies, Berkshire, Great Britain), pCAG-FLAG [74] (controls); the Tax-1 wildtype expression vectors pc-Tax [73], pSG5-Tax [75], pCAG-FLAG-Tax [74], pLcXL [76]; the vectors expressing Tax mutant proteins pc-M47 (CREB deficient), pc-M22 (NF-κB deficient), pc-M7 (CREB and NF-κB deficient), as described before [51]; the expression vector for an NF-κB deficient Tax-1 mutant pSG5-Tax-K1-10R [72]; the dominant negative inhibitor of IκBα expressing vector pIκBα-DN [88]; the vector expressing an IKK2 constitutive active mutant pcFLAG-IKK2-EE (IKK2-EE; [71]); the luciferase-reporter control vector pGL3-Basic (Promega, Mannheim, Germany), luciferase-reporter vectors harboring the *U3R* sequence of the HTLV-1 LTR pGL3-U3R-Luc (U3R-Luc; [63]) or harboring five NF-κB-responsive elements pNF-κB-Luc (NF-κB-Luc, Stratagene). The cDNAs of Tax wildtype, M47, M22 and M7 of pc-Tax, pc-M47, pc-M22 and pc-M7, respectively, and additionally of Tax S113A [48], were cloned into pEF-1α empty vector using standard cloning techniques and the restriction enzymes *BamHI* and *NotI* resulting in the Tax expression vectors pEF-Tax1, pEF-M7, pEF-M22, pEF-M7 and pEF-S113A.

#### Transfection of T-cells

For transient expression of Tax, 10×10^6^ or 5×10^6^ Jurkat, CCRF-CEM or Molt-4 T-cells were transfected with a total amount of 100 µg or 50 µg DNA, respectively. To generate RNA and Western Blot lysates, cells were separated before harvest where necessary. For luciferase reporter assays, 5×10^6^ Jurkat T-cells were co-transfected with 30 µg Tax mutant expression plasmid and 20 µg of the respective reporter vector. T-cells were electroporated with the *Gene Pulser X Electroporation System* (BioRad) at 290 V and 1500 μF.

#### Transfection of HEK-293 T cells

For transient expression experiments, 5×10^5^ 293 T cells were seeded in 6 Well plates 24 h prior to transfection. Transfection was conferred using *GeneJuice*® transfection reagent (Merck Millipore, Darmstadt, Germany) according to the manufacturer’s protocol using a total amount of 2 μg DNA. For luciferase reporter assays, 2×10^5^ 293 T cells were seeded in 12 Well plates and co-transfected 24 h later with a total amount of 1 µg DNA: 50 ng Tax expression plasmid and 100 ng of the respective reporter plasmid, replenished with pcDNA empty vector DNA.

### Quantitative real-time RT-PCR (qPCR)

At 48 h after transfection or inhibitor treatment, total cellular RNA was extracted (*NucleoSpin*® *RNA*, Macherey Nagel, Düren, Germany) and reversely transcribed to cDNA using *random hexamer primers* and *Superscript™* *II Reverse Transcriptase* (both Thermo Fisher Scientific, Waltham, MA, United States) according to the manufacturers’ instructions. 200 ng of cDNA was subjected to quantitative real-time RT-PCR (qPCR) applying *TaqMan*® *Universal PCR Master Mix* and an *ABI Prism 7500 Sequence Analyzer* (both Applied Biosystems, Foster City, CA, United States) according to the manufacturers’ instructions. Primer sequences and FAM (6-carboxyfluorescein)/TAMRA (tetramethylrhodamine)-labeled probes for detection of β*-actin* (ACTB) and *Tax* transcripts have been described before [99]. *Tax* transcripts were additionally detected using the probe 5′-FAM-TACAAGGCGACTGGTGCC-TAMRA-3′. Standard curves were generated for *Tax* by pEF-Tax1 expression plasmid or for *ACTB* by pJET1.2/blunt plasmid (Fermentas, St.-Leon Roth, Germany) bearing a respective *ACTB* target sequence to compute transcript expression levels. The mean of technical triplicates was calculated for all samples. Every experiment was independently performed at least three times and relative copy numbers (rcn) were calculated by normalization of respective transcript levels on those of β*-actin*.

### Western blot

For protein isolation, cells were resuspended at the indicated timepoints after transfection [48 or 72 h] in lysis buffer [150 mM NaCl, 10 mM Tris/HCl (pH 7.0), 10 mM EDTA, 1% Triton™ X-100, 2 mM DTT and protease inhibitors leupeptin, aprotinin (20 μg/ml each) and 1 mM phenylmethylsulfonyl fluoride (PMSF)], subjected to repeated *freeze-and-thaw* cycles between − 196 °C (liquid nitrogen) and 30 °C and additionally sonicated three times for 20 s. Equal amounts of proteins (between 30 and 50 μg) were denatured for 5 min at 95 °C in sodium dodecyl sulfate (SDS) loading dye (10 mM Tris/HCl (pH 6.8), 10% glycerin, 2% SDS, 0.1% bromophenol blue, 5% β-mercaptoethanol). SDS-PAGE and immunoblot using *Immobilon*®*-FL* PVDF transfer membranes (Merck Millipore, Billerica, MA, United States) were performed using standard protocols. Proteins were detected with the following primary antibodies: rabbit monoclonal anti-NF-κB2 p100/p52 (18D10, Cell Signaling Technologies), mouse anti-Tax (derived from the hybridoma cell line 168B17-46–34, provided by B. Langton through the AIDS Research and Reference Reagent Program, Division of AIDS, NIAID, NIH; [125]), mouse monoclonal anti-FLAG (M2, Sigma), rabbit polyclonal anti-IKK2 (Cell Signaling Technologies), mouse monoclonal anti-IκBα (3D6C02, Biolegend), mouse monoclonal anti-Hsp90 α/β (F-8, Santa Cruz Biotechnology), mouse monoclonal anti-α-Tubulin (T9026, Sigma), mouse monoclonal anti-ß-actin (AC-15, Sigma) and mouse monoclonal anti-GAPDH (3B1E9, GenScript). Secondary antibodies were anti-mouse or anti-rabbit Alexa Fluor® 647 (Life Technologies GmbH). Where necessary, antibodies were removed from the membrane by *Re-Blot Plus Strong Antibody Stripping Solution* (Merck Millipore) according to the manufacturer’s instructions, followed by subsequent staining steps. Fluorescence signals were assessed using the Advanced Fluorescence Imager camera (ChemoStar, Intas Science Imaging GmbH, Göttingen, Germany). Densitometric analysis was performed with the Advanced Image Data Analyzer (AIDA Version 4.22.034, Raytest Isotopenmessgeräte GmbH, Straubenhardt, Germany) to compare Tax or p100 expression levels, respectively.

### Treatment of transfected cells with chemical compounds

In experiments inducing NF-κB activity, transfected Jurkat T-cells were either treated 48 h after transfection with 20 ng/ml TNF-α (ImmunoTools GmbH, Friesoythe, Germany) for 15 min, 60 min or 24 h, respectively. Alternatively, cells were treated 24 h after transfection with 20 nM 12-O-Tetradecanoylphorbol-13-Acetate (TPA; Cell Signaling Technology) together with 1 µM ionomycin (Calbiochem, Merck Millipore) for another 24 h. In experiments repressing NF-κB activity, transfected Jurkat T-cells were treated 4 h after transfection with 2.5 µM 2-Amino-6-(2-(cyclopropylmethoxy)-6-hydroxyphenyl)-4-(4-piperidinyl)-3-pyridinecarbonitrile (ACHP, Merck Millipore) or the solvent control DMSO for 44 h. Moreover, Tax-transformed Tesi cells were treated for 48 h with 1, 2.5 or 5 µM ACHP or the solvent control DMSO, respectively. In experiments examining protein stability, in quadruplicate transfected Jurkat T-cells were pooled 24 h after transfection and separated into equal portions. Cells were subsequently treated for indicated periods of time with 50 µg/ml of the protein translation inhibitor cycloheximide (CHX, Sigma). In experiments fostering Tax protein expression, transfected Jurkat T-cells were treated 24 h after transfection with 20 mM of the lysosome inhibitor Ammonium chloride (NH_4_Cl; Sigma; dissolved in H_2_O), with 15 µM of the ubiquitin-activating enzyme E1 inhibitor 4[4-(5-nitro-furan-2-ylmethylene)-3,5-dioxo-pyrazolidin-1-yl]-benzoic acid ethyl ester (PYR-41; Sigma) or with the solvent control DMSO for 24 h.

### Luciferase reporter assays

Jurkat T-cells or 293 T cells were transfected as described above. 48 h after transfection, triplicate samples were processed to determine firefly luciferase activity as described before [63]. Relative light units (RLU) obtained from U3R-Luc or NF-κB-Luc were normalized on protein content and on background activity, which was assessed by the negative control vector pGL3-Basic.

### Statistics

For statistical analysis, two-tailed Student’s *t*-test was applied using Microsoft Excel. *p*-values below 0.05 or 0.01 were considered as significant (*) or highly significant (**), respectively.

## Supplementary information

**Additional file 1: Figure S1.** NF-κB deficient Tax mutants are functional and expressed on equal protein levels in HEK-293T cells.

**Additional file 2: Figure S2.** Expression of the NF-κB deficient Tax mutant M22 is rescued by co-expression of IKK2-EE in CCRF-CEM and Molt-4 T-cells.

**Additional file 3: Figure S3.** Expression of Tax and the NF-κB deficient Tax mutant M22 is not altered by induction of NF-κB signaling with TNF-α.

**Additional file 4: Figure S4.** NF-κB activity is important for Tax protein expression.

## Data Availability

Data sharing is not applicable to this article as no datasets were generated or analyzed during the current study.

## Abbreviations

ACHP: 2-Amino-6-(2-(cyclopropylmethoxy)-6-hydroxyphenyl)-4-(4-piperidinyl)-3-pyridinecarbonitrile
ATL/L: Adult T-Cell Leukemia/ Lymphoma
CHX: Cycloheximide
CREB: CAMP response element-binding protein
EBV: Epstein-Barr virus
HAM/TSP: HTLV-1-associated Myelopathy/ Tropical Spastic Paraparesis
HTLV-1: Human T-cell leukemia virus type 1
IκB: Inhibitor of kappa B
IKK: Inhibitor of kappa B kinase
LMP1: Latent Membrane Protein 1
LTR: Long terminal repeat
NEMO: NF-κB essential modulator
NFAT: Nuclear factor of activated T-cells
NF-κB: Nuclear factor kappa-light-chain-enhancer of activated B-cells
NIK: NF-κB inducing kinase
PMA: Phorbol 12-myristate 13-acetate
STAT3: Signal transducer and activator of transcription 3
SUMO: Small ubiquitin-related modifier
TAK: Transforming growth factor beta-activated kinase
Tax: Transactivator of the pX region
TNF-α: Tumor necrosis factor alpha
TPA: 12-O-Tetradecanoylphorbol-13-Acetate
