# Supplementary figures and images for: A novel positive feedback-loop between the HTLV-1 oncoprotein Tax and NF-κB activity in T-cells
Source: Retrovirology. 2020 Sep 10;17:30. doi: 10.1186/s12977-020-00538-w (PMC7488018; doi:10.1186/s12977-020-00538-w)

**A**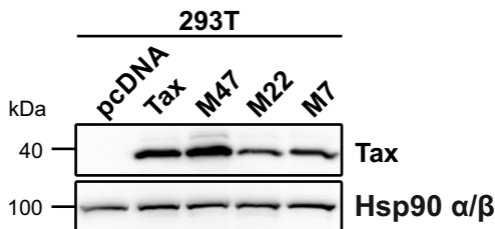**B**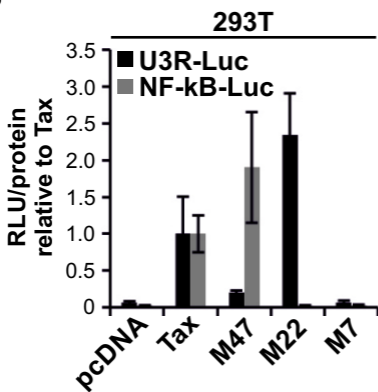**C**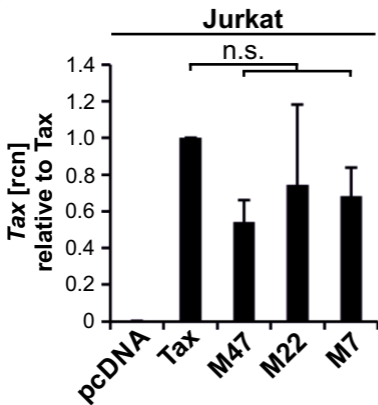

Supplement: Supplementary file 1 — Additional file 1: Figure S1. NF-κB deficient Tax mutants are functional and expressed on equal protein levels in HEK-293T cells. [file 12977_2020_538_MOESM1_ESM.pdf]

Supplementary Figure 2

Millen *et al.*

**A**

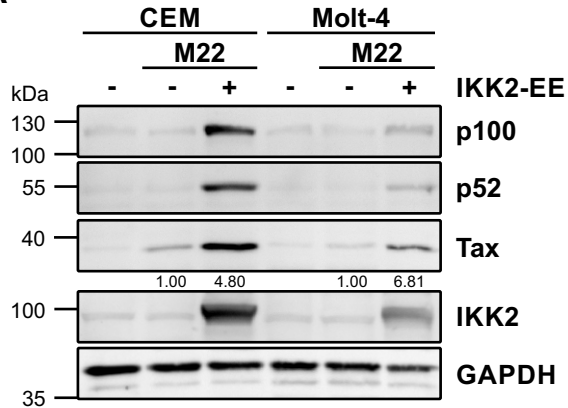

**B**

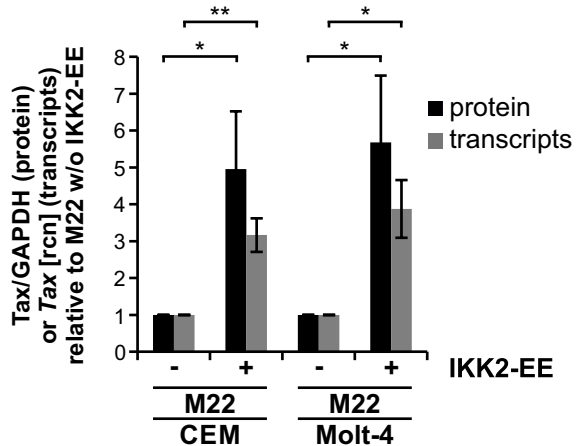

Supplement: Supplementary file 2 — Additional file 2: Figure S2. Expression of the NF-κB deficient Tax mutant M22 is rescued by co-expression of IKK2-EE in CCRF-CEM and Molt-4 T-cells. [file 12977_2020_538_MOESM2_ESM.pdf]

**A**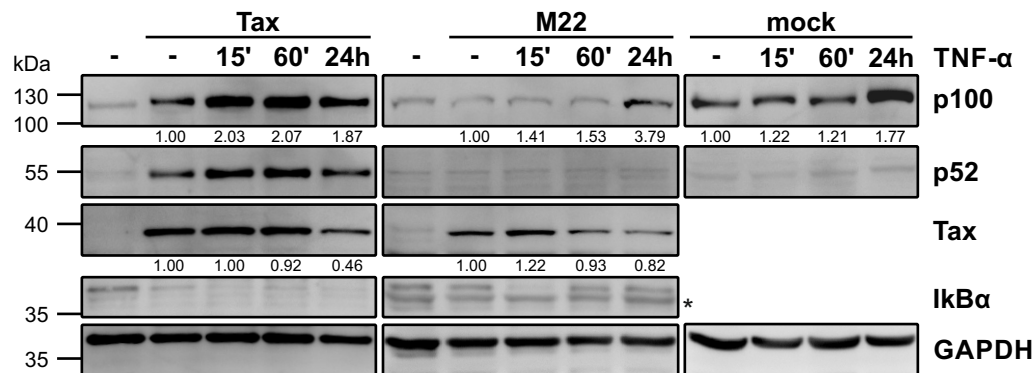**B**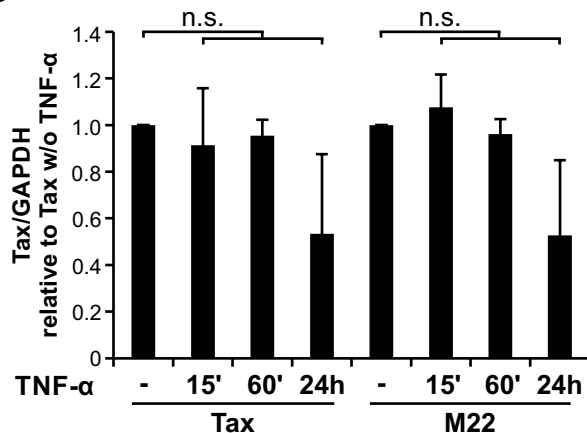**C**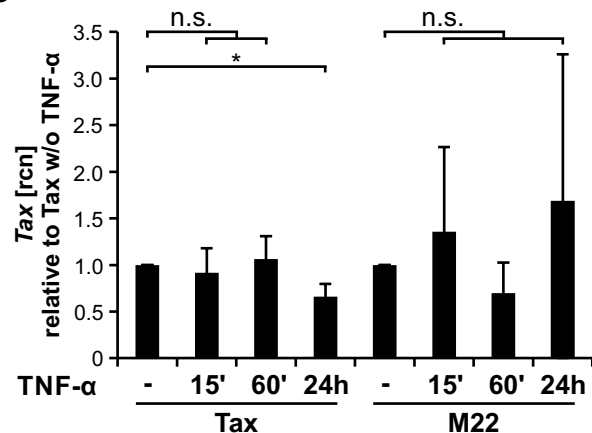

Supplement: Supplementary file 3 — Additional file 3: Figure S3. Expression of Tax and the NF-κB deficient Tax mutant M22 is not altered by induction of NF-κB signaling with TNF-α. [file 12977_2020_538_MOESM3_ESM.pdf]

# Supplementary Figure 4

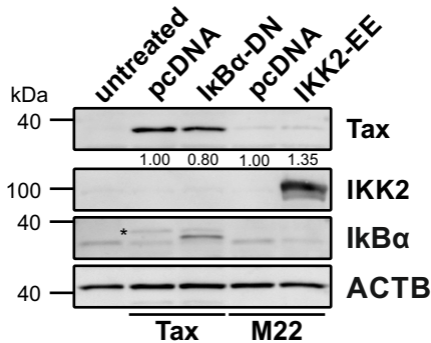

Supplement: Supplementary file 4 — Additional file 4: Figure S4. NF-κB activity is important for Tax protein expression. [file 12977_2020_538_MOESM4_ESM.pdf]
